# Biomimetic Nanotechnology Overcoming the Blood–Testis Barrier for Testicular Protection in Chemotherapy

**DOI:** 10.34133/bmr.0314

**Published:** 2026-01-30

**Authors:** Chaoli An, Jiefeng Sun, Ao Ma, Qi Mei, Bixiao Liu, Li Lu, Yu Yang, Wen Yu, Tao Song, Qingqiang Gao, Liang Shi, Qiuling Yue, Hui Wei, Xiaozhi Zhao

**Affiliations:** ^1^ Department of Andrology, Nanjing Drum Tower Hospital, The Affiliated Hospital of Nanjing University Medical School, Nanjing 210008, China.; ^2^ College of Engineering and Applied Sciences, Nanjing National Laboratory of Microstructures, Jiangsu Key Laboratory of Artificial Functional Materials, Nanjing University, Nanjing 210023, China.

## Abstract

Cancer patients exposed to chemotherapeutic drugs and whole-body radiation can result in testicular injury and germ cell loss. One of the mechanisms is that these drugs lead to the accumulation of reactive oxygen species (ROS) in the testes, which has been documented to cause testicular damage. Therefore, this highlights the critical need for ROS clearance in testes to preserve male fertility during cancer treatment. The blood–testis barrier (BTB) poses a major challenge, due to the absence of effective pharmaceutical agents that can penetrate this barrier to neutralize ROS effectively. We synthesized nanomaterials based on manganese-superoxide dismutase (PCN-222-Mn), demonstrating the ability to cross BTB and facilitate ROS clearance. Real-time T1-weighted magnetic resonance imaging confirmed the targeted delivery of PCN-222-Mn to the testes in mice. In murine models of testicular injury induced by cyclophosphamide, PCN-222-Mn showed major therapeutic effects by protecting germ cells and associated somatic cells through ROS reduction and autophagy enhancement. Additionally, PCN-222-Mn was demonstrated to penetrate Sertoli cells via clathrin-mediated and caveolae-mediated endocytosis and expelled by exocytosis, facilitating transport across the BTB. This research not only proposes a viable therapeutic approach to preserve male fertility during cancer treatment but also underscores the transformative potential of nanozymes in clinical settings.

## Introduction

Fertility protection is a hotspot in reproduction research, while cancer patients are important objects of fertility protection. Cancer patients exposed to chemotherapeutic drugs and whole-body radiation can result in testicular injury and germ cell loss. Nowadays, managing reproductive dysfunction in oncological patients primarily relies on sperm cryopreservation. For postpubertal patients, while sperm cryopreservation is available, it has limitations including decreased sperm quality and limited duration for cryopreservation. However, this approach is not feasible for prepubertal boys with cancer who have not yet begun spermatogenesis, leaving this vulnerable population without effective fertility preservation options. These limitations underscore the urgent need for alternative approaches to preserve male fertility during cancer treatment.

Cyclophosphamide (CTX) is a commonly used chemotherapeutic drug in clinical treatment. However, CTX was proved to be toxic in the male reproductive system [1], mainly affected spermatogenesis, and induced apoptosis of spermatogonia [2,3]. Moreover, CTX treatment could increase reactive oxygen species (ROS) concentrations while compromising antioxidant enzyme functionality [4], thus elevating levels of cellular oxidative imbalance. In physiologically normal circumstances, ROS serves essential functions in the acrosomal reaction and sperm capacitation [5]. However, an excessive amount of ROS can initiate lipid peroxidation in germ cells, resulting in genomic instability, compromised sperm mobility, and disrupted spermatogenesis, ultimately compromising male fertility [6].

The therapeutic potential of natural antioxidative enzymes is severely hampered by several inherent limitations, despite their demonstrated efficacy in ROS elimination through catalytic mechanisms. Key drawbacks include their poor stability, high production costs, and a lack of traceability in vivo. To address these challenges, nanozymes have been developed as a novel class of artificial enzymes. These functional nanomaterials, which possess enzyme-like activities, are engineered to overcome the shortcomings of their natural counterparts. In addition to the aforementioned aspects, nanozymes are amenable to precise tracing through the application of labeling and modification methodologies. They are particularly notable for their affordability, enhanced stability, versatility, and scalability [7,8].

Drug capacity to penetrate the blood–testis barrier (BTB) represents critical importance in managing testicular-related diseases. This barrier is an anatomical barrier separating the testicular vascular networks and the seminiferous tubules, primarily established through intercellular tight junction complexes among Sertoli cells within these tubular structures [9]. These cells protect developing germ cells from the external environment through intercellular junction complexes. However, this inherent defense mechanism also makes it difficult for many drugs to effectively enter testicular tissue, thereby affecting the therapeutic effect [10,11]. Therefore, identifying and evaluating BTB carrier-mediated drug delivery pathways is crucial for the successful treatment of these diseases. With the continuous advancement of nanoscale technology, its application scope has progressively expanded. Notably, recent studies have reported that nanozymes possess the capability to traverse the blood–brain barrier [12]. Consequently, exploring antioxidant nanomedicines that target the testes, especially those capable of crossing the BTB, will become a promising therapeutic avenue.

**Fig. 1.**
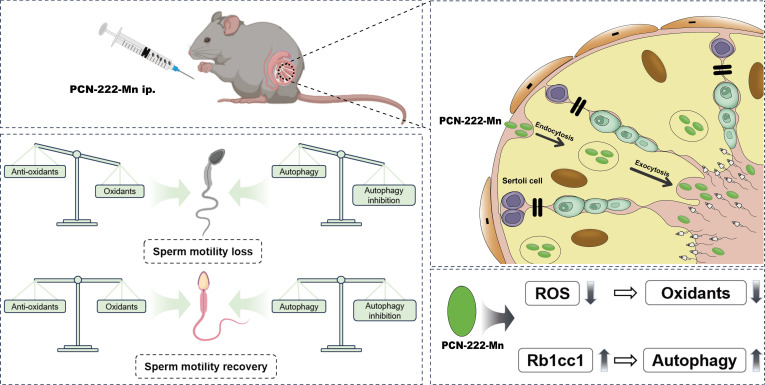
Graphical abstract of antioxidant nanozymes penetrating the blood–testis barrier and treating testicular injury.

In this study, we synthesized PCN-222-Mn, a nanozyme with superoxide dismutase (SOD)-like activity, by incorporating Mn(III) porphyrin units that mimic natural SOD metal active centers into the nanoscale zirconium-based metal-organic framework (MOF) PCN-222. The nanozymes demonstrated ROS-scavenging activity in vivo and localized within the testes (Fig. 1). This strategy effectively recovered CTX-induced testicular injury in mice, offering a novel therapeutic approach for its clinical management.

## Materials and Methods

### Materials

TM3/TM4/GC1 cells were purchased from the Cell Bank of the Chinese Academy of Sciences (Shanghai, China). Fetal bovine serum (FBS), penicillin–streptomycin, dichlorofluorescin diacetate (DCFH-DA), AceQ qPCR SYBR Green Master Mix, diaminobenzidine (DAB), TRIzol reagent, and the PrimeScript RT Reagent Kit were sourced from Wisent (Nanjing, China), Gibco (Massachusetts, USA), Beyotime Biotech (Shanghai, China), Vazyme Biotech (Nanjing, China), ZSGB-BIO (Beijing, China), Invitrogen (Carlsbad, USA), and Takara (Otsu, Japan), respectively. Primary antibodies used for immunohistochemistry (IHC) were as follows: anti-GATA1 (Proteintech, 10,917-2-AP, 1:1,000), anti-3β-HSD (Proteintech, 67,572-1-AP, 1:1,000), and anti-DDX4 (Abcam, ab270534, 1:2,000). The antibodies used for immunofluorescence staining were as follows: ZO-1 (Abcam, ab276131, 1:2,500) and OCCLUDIN (Abcam, ab216327, 1:2,500). Quant-iT Pico Green dsDNA reagent was from Thermo Fisher Scientific (Shanghai, China). Chlorpromazine (CPZ), Wortmannin (Wort), and Brefeldin A (BFA) were obtained from MedChemExpress (Shanghai, China). Nystatin (Nys) was acquired from Beyotime Biotech (Shanghai, China). 7-Ketocholesterol (7-KC) was sourced from TargetMol (Shanghai, China).

### Synthesis of TCPP-Mn

The preparation of TCPP-Mn proceeded through the following protocol. TPP-COOMe (0.854 g) combined with 2.5 g MnCl_2_·4H_2_O (molar ratio of porphyrin to MnCl₂ is 1:12.5) were solubilized in 100 ml in N,N′ dimethylformamide (DMF) solvent and subjected to reflux heating for 6 h under light-free conditions. H_2_O (150 ml) was introduced upon cooling the mixture to ambient temperature, followed by continued stirring for 60 min. Solid separation occurred via filtration, with subsequent washing using distilled water in triplicate. The recovered solid underwent additional filtration and triple washing with H_2_O. After washing, this resulting dark-greenish crystalline material was subsequently solubilized in CHCl_3_ and purified with H_2_O. Target molecule synthesis required dissolving 0.75 g of the crystalline product using a 3-component solvent mixture (MeOH 25 ml, THF 25 ml, H_2_O 25 ml, and KOH 2.63 g), followed by 12-h reflux in darkness. THF and MeOH were eliminated via rotary evaporation, subsequently followed by water addition and 1 M HCl introduction to achieve complete acidification of the precipitate. After filtration and drying in vacuum, TCPP-Mn was successfully prepared.

### Synthesis of nanoscale PCN-222-Mn

Nanoscale PCN-222-Mn was synthesized following a reported procedure with experimental adjustments [13]. Generally, 20 mg of TCPP-Mn, 60 mg of ZrOCl_2_·8H_2_O, and 580 mg of benzoic acid were dissolved in 20 ml of DMF, and magnetically stirred (300 rpm) at 90 °C for 5 h. After the solution cooled to room temperature, PCN-222-Mn NPs were obtained by centrifugation (15,000 rpm, 30 min) and washed with DMF for several times. The resulting PCN-222-Mn NPs were redispersed in DMF for storage.

### Characterization of nanoscale PCN-222-Mn

The nanoscale PCN-222-Mn was observed under a transmission electron microscope (Tecnai F20 microscope FEI, Field Electron and Iron Company) at an acceleration voltage of 200 kV and a scanning electron microscope (Zeiss Ultra 55 microscope, Zeiss, Germany). Powder x-ray diffraction (PXRD) analysis was conducted using an ARL SCINTAC X’TRA diffractometer employing Cu Kα radiation (Thermo Fisher Scientific). Optical absorption characteristics of samples were determined via ultraviolet–visible (UV–Vis) spectrophotometry utilizing a 1-cm quartz cuvette (Purkinje General Instrument Co. Ltd., Beijing, China) and microplate detection system (SpectraMax M2e, Molecular Device Co. Ltd., Shanghai, China). Surface charge properties of nanoscale PCN-222-Mn were determined using a Nanosizer ZS90 (Malvern).

### Determination of SOD-like activity of PCN-222-Mn

To measure SOD activity using the hydroethidine (HE) method, the following solutions were prepared: HE solution (2 mg/ml in DMSO), xanthine solution (15 mM), xanthine oxidase solution (0.84 U/ml), and Tris-HCl buffer (pH 7.4). PCN-222-Mn was used at concentrations of 200 μg/ml, 400 μg/ml, 600 μg/ml, 800 μg/ml, and 1 mg/ml. The assay was performed by adding 100 μl of HE solution, 650 μl of Tris buffer, 100 μl of xanthine solution, and 100 μl of the material solution in sequence to a brown test tube. Finally, 50 μl of xanthine oxidase solution was added, and the mixture was incubated at 37 °C for 30 min in the dark. Fluorescence readings were then taken using a fluorometer with an excitation wavelength of 470 nm and a scanning range of 500 to 700 nm.

### Determination of CAT-like activity of PCN-222-Mn

Catalase (CAT) activity was determined using a dopamine-based colorimetric assay. Briefly, 100 μl of dopamine solution (2 mg/ml) was added to each well of a microplate and placed in an anaerobic bag to remove oxygen. Subsequently, 10 μl of H_2_O_2_ (100 mM), 4 μl of material solution (PCN-222-Mn at concentrations of 250 μg/ml, 500 μg/ml, 1 mg/ml, or 2 mg/ml, or TCPP-Mn at 2 mg/ml), and 86 μl of Tris-HCl buffer (pH 8.5) were rapidly added to minimize atmospheric oxygen interference. The reaction mixture was incubated at 37 °C for 10 to 15 min, followed by absorbance measurement at 405 nm using a microplate reader. All solution additions were performed as quickly as possible to reduce potential interference from atmospheric oxygen.

### Determination of the POD/OXD-like activity of PCN-222-Mn

Peroxidase (POD)-like and oxidase (OXD)-like activities of PCN-222-Mn were evaluated by TMB oxidation, monitoring absorbance at 652 nm. Reactions (1 ml total volume) contained nanozyme suspension (30 μl, 10 mM), TMB (50 μl, 10 mM), and PBS (820 μl for POD, 920 μl for OXD; 0.02 M, pH 7.2). For POD activity, H_2_O_2_ (100 μl, 10 mM) was included. For OXD activity, H_2_O_2_ was omitted. After 20 min of incubation at room temperature, absorbances were recorded using a UV–Vis spectrophotometer.

### In vivo MRI of testes

The in vivo accumulation of PCN-222-Mn in murine testes was monitored employing a 9.4-T Bruker BioSpec 94/20 MR system (Bruker BioSpin MRI, Ettlingen, Germany). For the procedure, mice were first anesthetized via isoflurane inhalation to acquire baseline (0 h) magnetic resonance imaging (MRI) scans. Subsequently, each mouse received an injection of PCN-222-Mn (1 mg/kg) and was subjected to further MRI scanning at 12, 24, 48, and 72 h after injection. All acquired images received analysis via ITK-SNAP software for subsequent signal quantification.

### In vivo biocompatibility of PCN-222-Mn

Healthy male C57BL/6 mice were continuously intraperitoneally injected with 1 mg/kg PBS or PCN-222-Mn for 7 days. Then, major organs (heart, liver, spleen, lung, kidney, and testis) were harvested for hematoxylin and eosin (H&E) analysis.

### Animals

The C57BL/6J mice (6 to 8 weeks old, 20 to 23 g, male, specific pathogen free) were housed in the Animal Center of the Affiliated Drum Tower Hospital of Nanjing University Medical School. All mice experiments were performed in accordance with the requirements of the Nanjing Drum Tower Hospital Laboratory Animal Guidelines. Animal experimental protocols received approval and licensing from the Ethics Committee of Nanjing Drum Tower Hospital (2021AE01035).

### CTX-induced testicular injury mouse model

The testicular injury mouse model was established by intraperitoneal injection with CTX. Generally, male C57BL/6 mice (6 to 8 weeks) were divided into 3 groups: NC, CTX, and CTX + PCN-222-Mn (*n* = 6 per group, detailed sample size calculation is provided in Table S1). Mice were intraperitoneally injected with PBS (NC group) or CTX (CTX and PCN-222-Mn groups) with a dosage of 50 mg/kg for 7 days. After the continuous intraperitoneal injection of CTX or PBS, mice were further intraperitoneally injected with PBS (NC and CTX groups) or PCN-222-Mn (CTX + PCN-222-Mn group) with a dosage of 1 mg/kg for another 7 days. Then, the mice were harvested to evaluate the therapeutic efficacy of PCN-222-Mn.

### Analyses of mouse sperm concentration and motility

Mice were sacrificed, and epididymis were collected. The cauda epididymis was cut and placed in Dulbecco’s Modified Eagle Medium (DMEM) culture medium enriched with 10% FBS under 37 °C incubation conditions for 5 min. Then, the liquid containing sperm was collected, and sperm concentration and motility were evaluated through Computer-Assisted Sperm Analysis methodology.

### Assessment of testosterone level

After mice were sacrificed, blood specimens were collected and treated with heparin anticoagulant immediately. Serum was isolated through centrifugal separation of blood specimens at 3,000 rpm for 15 min at 4 °C. The Testosterone Enzyme-Linked ImmunoSorbent Assay Kit was used to measure testosterone level. Each assay was conducted according to manufacturer-supplied procedures.

### H&E and IHC staining of testis and epididymis sections

Testis and epididymis tissues were collected and preserved in paraformaldehyde solution (4% in PBS), processed for paraffin embedding, subsequently sectioned at 5 μm thickness and subjected to H&E or IHC staining procedures. Histological and IHC analyses were conducted according to established protocols, and antibodies used in IHC staining were described before. Representative microscopic images were acquired using a high-resolution microscope (×200 magnification) through a Leica LAS v4.12 imaging system.

### In vivo ROS-scavenging activity of PCN-222-Mn

Testicular tissues were preserved in Optimal Cutting Temperature compound embedding medium at −20 °C for cryosection preparation (5 μm). Cryopreserved testicular sections underwent treatment with 0.3% Triton X-100 during 15 min under ambient conditions and labeled with dihydroethidium (DHE) (1 mM) during 30 min. Subsequently, the sections received PBS rinsing 3 times and 4′,6-diamidino-2-phenylindole counterstaining. Fluorescent signals were visualized using a fluorescence microscope.

### Quantitative real-time polymerase chain reaction

TRIzol reagent enabled total testicular RNA extraction, with subsequent cDNA synthesis utilizing the PrimeScript RT Reagent Kit for reverse transcription protocols. PCR amplification employed AceQ qPCR SYBR Green Master Mix, while StepOnePlus instrumentation (Applied Biosystems, Foster City, USA) provided thermal cycling capability. Primers are listed in Table S2. ACTB served as the reference standard for relative mRNA quantification normalization.

### In vitro cytotoxicity of PCN-222-Mn

TM3/TM4/GC1 cell lines were maintained in DMEM supplemented with 10% FBS and 1% penicillin−streptomycin within a humidified environment containing 5% CO_2_ at 37 °C. For cytotoxicity assessment of PCN-222-Mn, cells were plated in 96-well plates at a density of 5 × 10^3^ cells per well and incubated for 24 h. Subsequently, the cells were exposed to PCN-222-Mn at various concentrations (0.16, 0.32, 0.63, 1.25, 2.5, 5, and 10 μg/ml) for additional 24- or 72-h periods. Cell viability was assessed using the 3-(4,5-dimethylthiazol-2-yl)-2,5-diphenyltetrazolium bromide (MTT) colorimetric assay.

### In vitro protective effect of PCN-222-Mn on testicular cells against PM

Cells were cultured as previously described. Testicular cells (TM3/TM4/GC1) were plated onto 96-well culture dishes at a concentration of 5 × 10^3^ cells per well for 24 h; subsequently, the cellular population underwent coexposure using PM (200 μM) for 24 h and rinsed with PBS; thereafter, cells received treatment with PCN-222-Mn in different concentrations for 24 h. MTT colorimetric analysis was employed to assess cellular viability.

### Intracellular ROS scavenging detection

The treatment of testicular cells was performed as previously described. After the treatment of PCN-222-Mn, spent culture medium underwent removal followed by triple PBS rinsing cycles. DCFH-DA (0.01 mM in FBS-free DMEM), a fluorogenic probe, was dispensed into individual wells and cultured for 30 min. Subsequently, medium underwent triple exchange procedures, and fluorescence intensities of DCFH-DA were measured using a Beckman CytoFLEX flow cytometer in real time.

### Western blot

For target protein quantification, testicular tissue-derived protein extracts underwent immunoblotting procedures following established methodologies [14]. Antibodies used in Western blot were described before.

### In vitro BTB model

TM4 cells were cultured with a density of 5 × 10^3^ cells per well for 24 h. Immunofluorescence staining of OCCLUDIN and ZO-1 was performed to confirm the construction of BTB. Then, TM4 cells were cultured in the same way in a transwell plate, and culture medium with PCN-222-Mn and inhibitors was added as described.

### Measurement of Mn content

The Mn content of the culture media was measured by inductively coupled plasma mass spectrometry (Agilent 7850 MS, Agilent, USA).

### Statistical analysis

Statistical analysis involved 2-sided Student’s *t* test for 2 groups and one-way analysis of variance (ANOVA) for multiple groups. *P* < 0.05 was considered statistically significant.

## Results

### Design, synthesis, and characterization of PCN-222-Mn

**Fig. 2.**
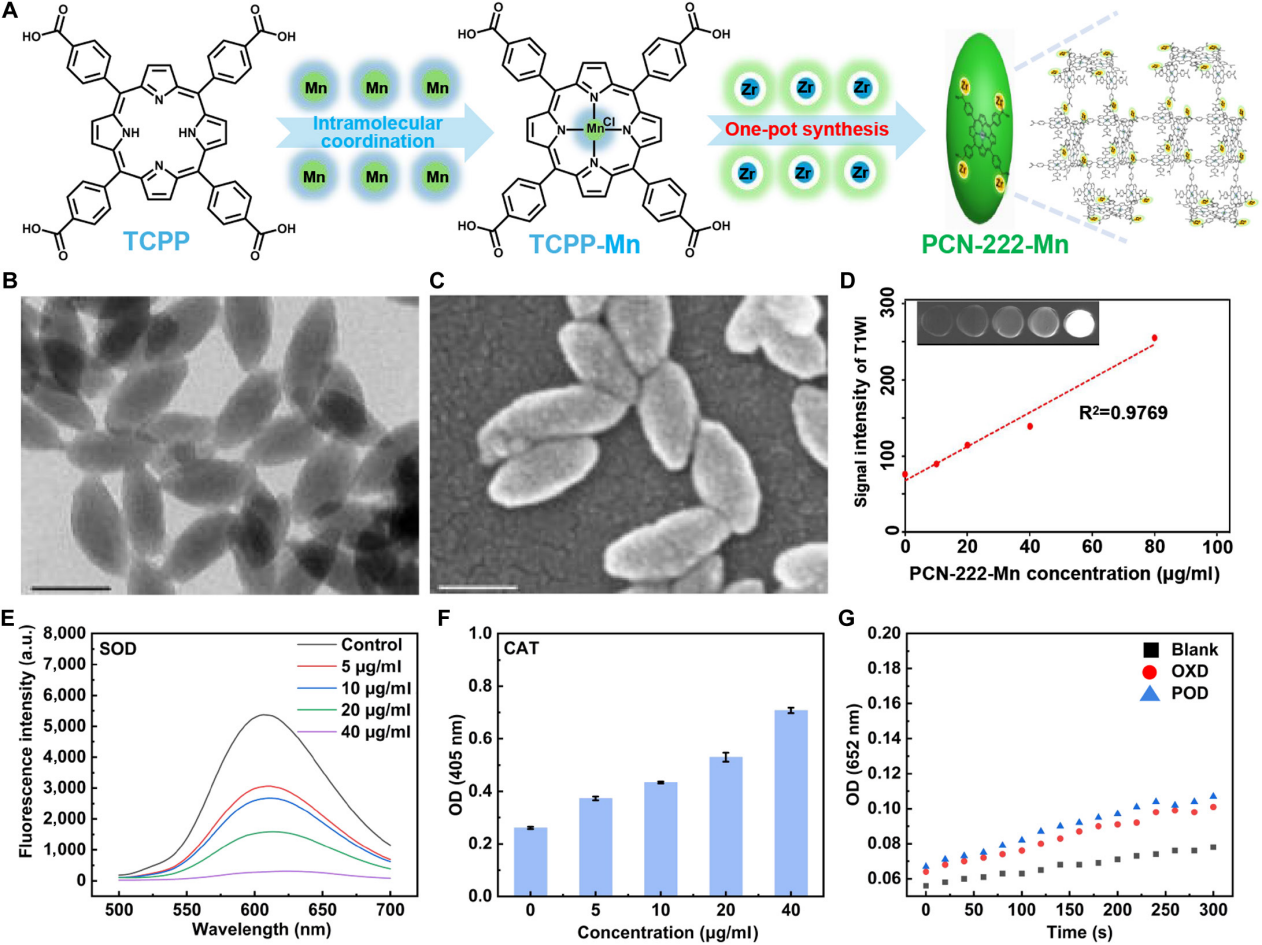
(A) Synthetic pathway for PCN-222-Mn fabrication. (B and C) Morphological characterization of PCN-222-Mn via transmission electron microscopy (TEM) and scanning electron microscopy (SEM). Scale bar: 100 nm. (D) MRI ability of PCN-222-Mn. (E to G) Multienzyme mimetic activities of PCN-222-Mn showing SOD (E), CAT (F), and OXD/POD (G)-like properties.

The design and synthesis of PCN-222-Mn were systematically executed as illustrated in Fig. 2A. Following established synthetic protocols [15], we first synthesized the manganese porphyrin [5,10,15,20-tetrakis(4-carboxyphenyl)porphyrinato]-Mn(III) chloride (TCPP-Mn), which structurally mimics the mononuclear Mn(III) active site in natural Mn-SOD [15]. Then, the nanoscale MOF containing TCPP-Mn, designated as PCN-222-Mn, was synthesized.

The successful synthesis of PCN-222-Mn was verified through multiple complementary characterization techniques. Spectroscopic characterization using UV–Vis spectroscopy confirmed the successful integration of TCPP-Mn into the framework, as evidenced by the distinctive absorption peaks at 470, 570, and 610 nm (Fig. S1A). Structural characterization through transmission electron microscopy (TEM) and scanning electron microscopy (SEM) revealed consistent lens-shaped nanostructures possessing mean dimensions of approximately 200 nm (Fig. 2B and C). Additionally, the crystalline structure was validated through PXRD analysis (Fig. S1B), which corresponded to previously documented data, further validating the successful formation of the intended MOF structure. Moreover, the nanozymes exhibited excellent dispersibility in aqueous solution with a zeta potential of approximately +28 mV (Fig. S1C), indicating promising potential for future in vivo applications without aggregation concerns [15].

Addressing a key limitation of conventional MOFs, which often prove challenging for in vivo tracking and biodistribution analysis, the incorporation of Mn ions into PCN-222-Mn confers distinct advantages for their detection and traceability in vivo. This enhancement is attributed to the well-established efficacy of Mn ions, particularly when nanostructured, serving as T1-weighted MRI contrast media, exemplified by manganese oxide nanoparticles (MONs) [16], Prussian blue nanoparticles, and encapsulated Mn^2+^ ions in sealed carbonized shells. In line with these properties, subsequent experimental evaluation revealed that following incubation in PBS for 72 h, PCN-222-Mn exhibited a bright T1 signal with an *R*^2^ value of 0.9769 (Fig. 2D), demonstrating its potential as a contrast agent for bioimaging.

We further investigated the antioxidant properties of PCN-222-Mn by assessing its mimetic activities for key enzymes, namely, SOD, CAT, OXD, and POD. PCN-222-Mn showed robust SOD-like activity in a clear concentration-dependent manner (Fig. 2E), indicating efficient scavenging of superoxide radicals and comparing favorably with recently reported materials [17,18]. In addition to its pronounced SOD-mimetic activity, PCN-222-Mn also exhibited moderate, dose-dependent CAT-like activity, as evidenced by a monotonic increase in the 405-nm absorbance associated with H_2_O_2_ decomposition (Fig. 2F). Conversely, no significant OXD/POD-like activities were observed for PCN-222-Mn (Fig. 2G). Our previous studies showed that PCN-222-Mn exhibits stronger SOD-like activity compared to free TCPP-Mn, suggesting an enhancement from the MOF framework [15]. Free metalloporphyrins like TCPP-Mn may be prone to aggregation in solution, potentially restricting substrate access to the Mn(III) center, while also exhibiting poor in vivo pharmacokinetics, including rapid clearance and limited targeting [19,20]. The PCN-222 structure appears to mitigate these issues by providing spatial separation to prevent stacking and facilitating ROS diffusion, as well as enhancing stability and biodistribution for better tissue accumulation. Based on these findings, we proceeded with subsequent cellular and animal experiments.

### Evaluation of PCN-222-Mn effects on testicular cells in vitro and its biodistribution and biocompatibility in vivo

**Fig. 3.**
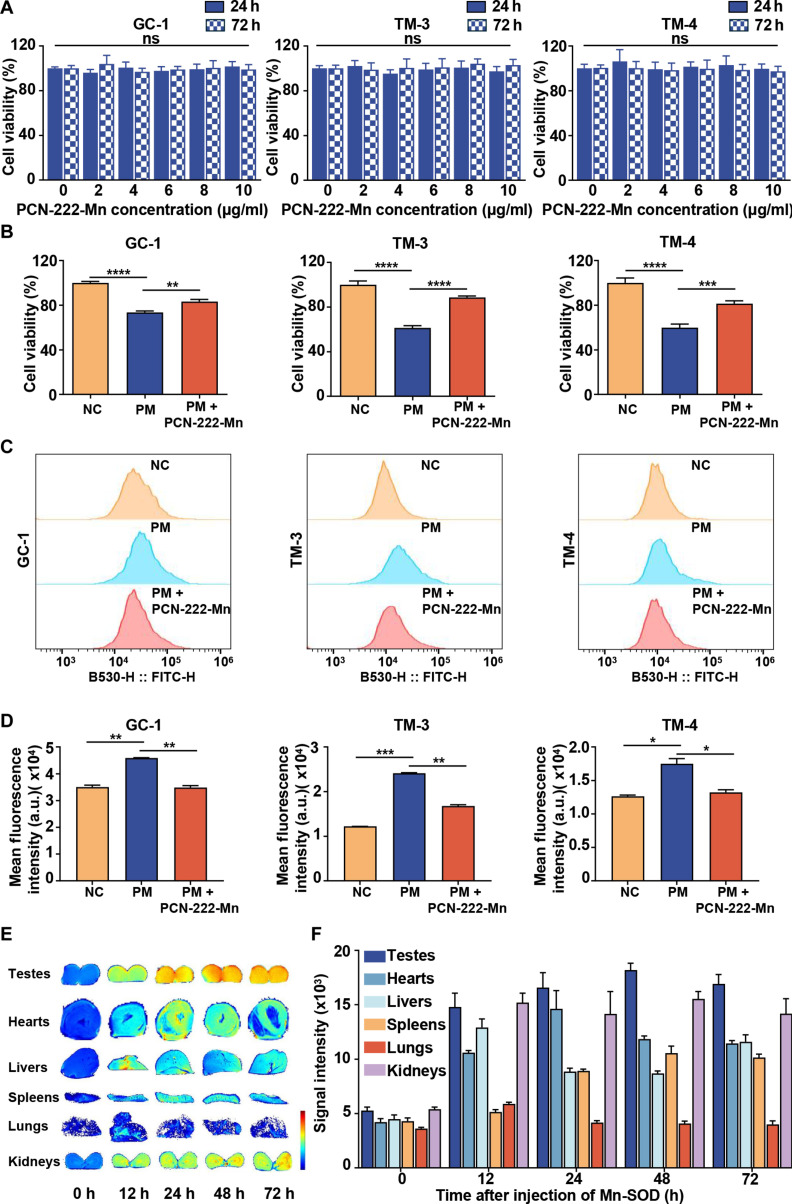
(A) Cell viability 24 or 72 h after coincubated with PCN-222-Mn on GC1, TM3, and TM4 cells. (B) Cell viability after coincubated with PM and PCN-222-Mn on GC1, TM3, and TM4 cells. (C) Antioxidative activities of PCN-222-Mn on GC1, TM3, and TM4 cells in vitro measured by flow cytometry in PM models. (D) Statistical analysis of antioxidative activities of PCN-222-Mn on GC1, TM3, and TM4 cells in vitro*.* Data are shown as mean ± SEM, 2-tailed Student’s *t* test for *P* values (*P < 0.05, **P < 0.01, ***P < 0.001, ****P < 0.0001). (E) Representative MR images of testes, hearts, livers, spleens, lungs, and kidneys at 9.4 T after PCN-222-Mn injection. (F) MRI signal intensity quantifications at 0, 12, 24, 48, and 72 h after injection. Data are presented as mean ± SEM (*n* = 3).

To detect the antioxidant activity of PCN-222-Mn on testicular cells, TM3 cells (Leydig cells), TM4 cells (Sertoli cells), and GC1 cells (spermatogonia cells) were utilized. Cell viability was determined through MTT colorimetric analysis. Phosphoramide mustard (PM), the active metabolite of CTX, was used to establish cell models for in vitro experiments, and the cytotoxicity of PCN-222-Mn was measured initially. Results indicated that PCN-222-Mn exhibited no significant cytotoxicity at concentrations up to 10 μg/ml after 24 and 72 h (Fig. 3A), demonstrating acceptable biocompatibility. The protective effect of PCN-222-Mn against PM treatment on testicular cells was then examined. The results revealed significant reductions in cell viability in TM3, TM4, and GC-1 cells following PM treatment, which was partially rescued by PCN-222-Mn (Fig. 3B). Furthermore, an increased ROS level was observed in all 3 cell lines post-PM treatment, whereas PCN-222-Mn treatment significantly reduced the ROS levels (Fig. 3C and D). Consequently, we concluded that PCN-222-Mn treatment could protect testicular cells from PM-induced ROS and improve cellular viability. To check the biodistribution and biocompatibility, PCN-222-Mn was administered intraperitoneally to adult male mice. Subsequently, T1-weighted MRI was performed at various time points (0, 12, 24, 48, and 72 h postinjection) to monitor changes in major organs. It revealed that PCN-222-Mn is predominantly distributed and enriched in the testes, with minimal distribution in other organs except kidneys, which are prone to drug accumulation due to their rich blood supply (Fig. 3E). PCN-222-Mn levels in the testes elevated gradually, remaining elevated until 72 h postinjection, which is beneficial for its function within the testes (Fig. 3F). To assess the biocompatibility of this nanomaterial, healthy mice received intraperitoneal administration of PCN-222-Mn (1 mg/kg for 7 days), and vital organs, encompassing the heart, liver, spleen, lungs, kidneys, and testes, were collected 24 h after the final injection. H&E histological analysis of the examined tissues showed no notable histological alterations (Fig. S2A), confirming excellent biocompatibility of this nanomaterial in vivo.

### Therapeutic efficacy of PCN-222-Mn in a CTX-induced testicular injury model in vivo

**Fig. 4.**
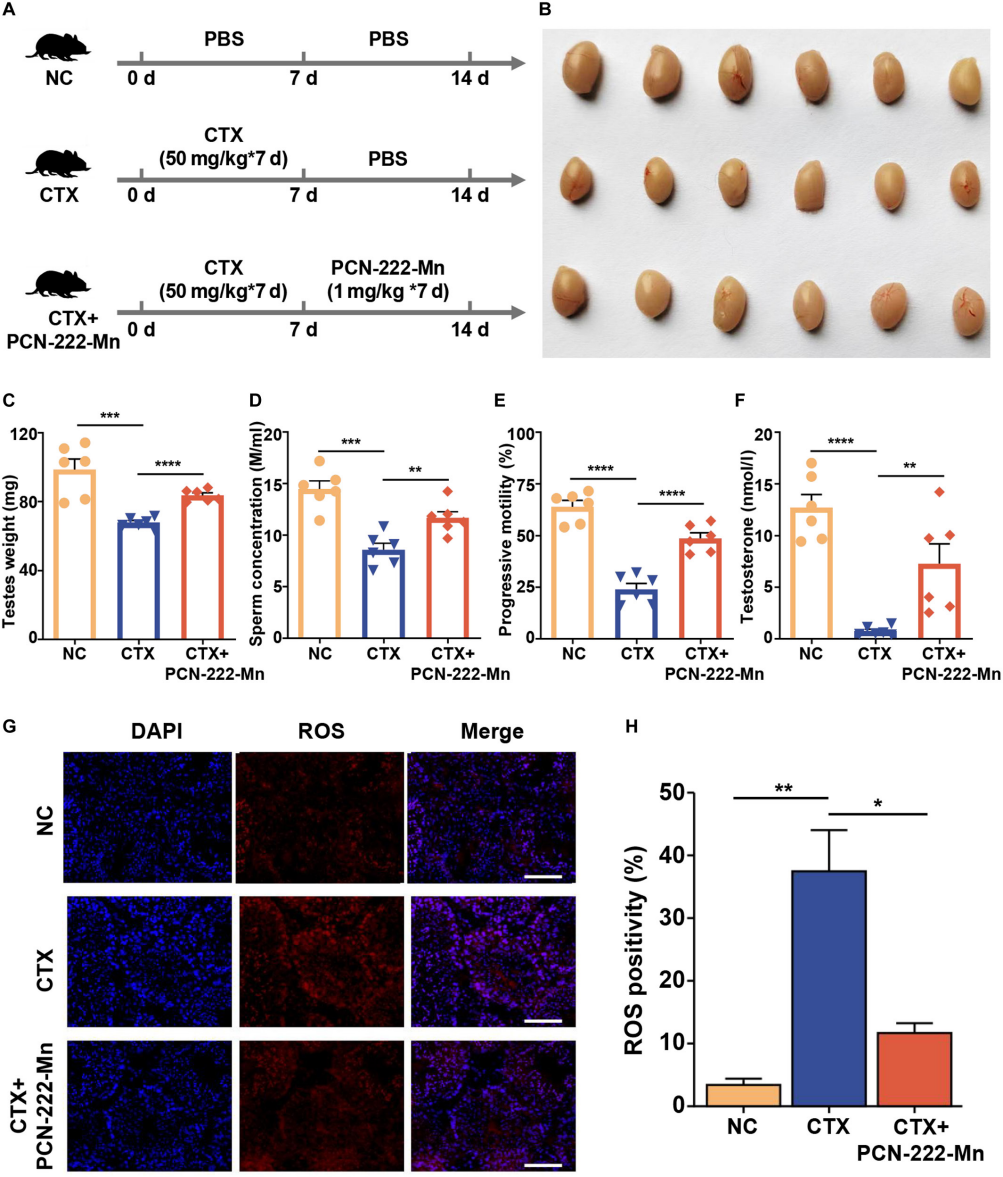
Therapeutic efficacy of PCN-222-Mn treatment in a CTX-induced testicular injury mouse model. (A) Diagrammatic overview depicting CTX-mediated testicular damage induction and subsequent PCN-222-Mn therapeutic intervention. (B to F) Testicular size (B), unilateral testicular weight (C), concentration (D), progressive motility (E) of sperm from cauda epididymis, and serum testosterone concentration (F) of mice from NC, CTX, and CTX + PCN-222-Mn groups. Data are shown as mean ± SEM (*n* = 6), 2-tailed Student’s *t* test for *P* values (**P < 0.01, ***P < 0.001, ****P < 0.0001). (G) DHE staining of testes sections from NC, CTX, and CTX + PCN-222-Mn groups. Scale bar: 100 μm. (H) Statistical analysis of ROS levels by intensity of fluorescence.

CTX-induced testicular injury mouse model development occurred through the intraperitoneal administration of CTX using 50 mg/kg body weight doses across 7 consecutive days, with PBS-treated mice serving as controls [21,22]. Subsequently, PCN-222-Mn intervention protocols employed 1 mg/kg body weight for 7 days, with PBS-treated mice serving as controls (Fig. 4A). To assess the therapeutic efficacy of PCN-222-Mn, testicular size and weight evaluation proceeded alongside sperm concentration, progressive motility, and serum testosterone level assessments. These parameters experienced significant reduction in the model group but improved following PCN-222-Mn treatment (Fig. 4B to F). H&E staining analysis indicated structural disarray among the seminiferous tubules of the model group, characterized by loss of germ cells, and enlarged gaps and lumens, accompanied by decreased epididymal sperm density. In contrast, PCN-222-Mn treatment markedly ameliorated these defects (Fig. S2B). Considering the established impact of ROS on sperm motility impairment [23], we assessed the production of O_2_^−^ in frozen testis sections. As anticipated, the CTX model group demonstrated substantially higher O_2_^−^ levels than the NC group.

### Effects of PCN-222-Mn on genes and proteins of testicular cells in vivo

To elucidate the molecular mechanisms underlying the therapeutic effects of PCN-222-Mn on testicular cells, we initially assessed the expression of related genes and proteins following PCN-222-Mn treatment. In CTX-treated mice testes, mRNA expression of inflammatory factors-related genes (*Tnf* and *Il1b*) was enhanced and Leydig cells-related genes (*Lhcgr*, *Hsd17b*, *Cyp11a1*, and *Star*) were reduced, consistent with CTX-induced testicular injury. Notably, PCN-222-Mn treatment partially reversed these CTX-induced alterations in mRNA expression, restoring them toward normal levels (Figs. S3A to D and S4). Furthermore, IHC staining demonstrated that, compared to the CTX model group, the CTX + PCN-222-Mn treated group exhibited significantly increased protein expression of 3β-HSD (a Leydig cell marker), GATA1 (a Sertoli cell marker), and DDX4 (a germ cell-specific protein) [24] (Fig. S3E). Overall, PCN-222-Mn treatment improved the gene and protein expression in several cell types critical for normal spermatogenesis, including Leydig cells, Sertoli cells, and germ cells. These findings demonstrate the therapeutic potential of PCN-222-Mn in the testicular injury model.

### PCN-222-Mn improves sperm motility in testicular injury mice through antioxidant and autophagy enhancement

**Fig. 5.**
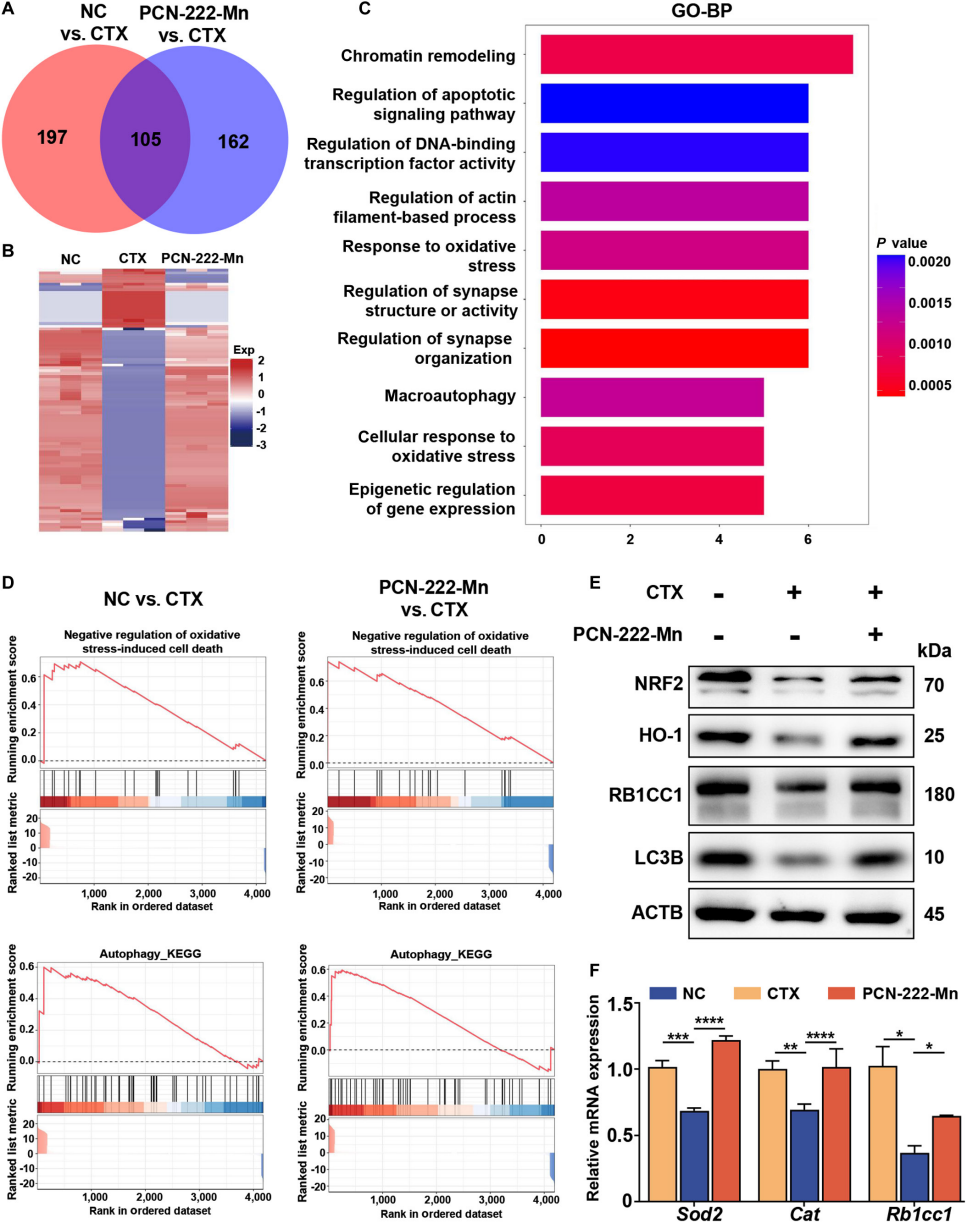
Proteomic analyses of PCN-222-Mn treatment for the CTX-induced testicular injury. (A) Venn diagram representing the set of all differentially expressed proteins (DEPs). (B) Heat map of the 105 shared DEPs. (C) GO pathway analysis of the shared DEPs. (D) Gene Set Enrichment Analysis (GSEA) result of differentially expressed proteins in “Negative regulation of oxidative stress-induced cell death” and “Autophagy”. (E) Western blot analysis for related proteins. (F) qPCR analysis for the related factors.

To further investigate the potential molecular mechanisms by which PCN-222-Mn exerts its therapeutic effects on testicular injury, proteomic analyses were conducted on mouse testes. A total of 4,035 proteins were identified, including 302 DEPs between the NC and CTX groups, and 267 DEPs between the CTX and PCN-222-Mn groups. Notably, 105 DEPs exhibited reverse expression changes in the PCN-222-Mn group compared to the CTX group, suggesting recovery following PCN-222-Mn treatment (Fig. 5A and B). Gene Ontology (GO) pathway analysis highlighted the primary affected pathways as apoptotic signaling, oxidative stress response, and autophagy (Fig. 5C).

Consistent with these findings, Gene Set Enrichment Analysis (GSEA) indicated that CTX treatment led to the dysregulation of pathways related to oxidative stress responses and autophagy. Specifically, pathways promoting antioxidant defense and beneficial autophagy appeared to be suppressed by CTX, and these detrimental changes were effectively reversed by PCN-222-Mn treatment (Fig. 5D).

To validate the expression of relevant proteins, quantitative reverse transcription polymerase chain reaction (qRT-PCR) was employed to measure the mRNA expression of *Sod2* and *Cat*, 2 genes related to antioxidant enzymes, in the testes. Western blot analysis was performed to determine the protein abundance of 2 classic antioxidant proteins, NRF2 and HO-1, in testicular tissues. Results showed that CTX treatment markedly decreased the mRNA and protein levels of these antioxidant-related genes/proteins, whereas PCN-222-Mn treatment restored their expression (Fig. 5E and F). Similarly, the autophagy pathway was found to be down-regulated following CTX treatment and was restored by PCN-222-Mn treatment. The expression of autophagy-related genes, *Rb1cc1* and *Lc3b*, was evaluated by qRT-PCR and Western blot, showing decreased expression after modeling and effective restoration following PCN-222-Mn treatment (Fig. 5E and F). These findings indicate that PCN-222-Mn can activate antioxidant signaling pathways and enhance autophagy, thereby mitigating CTX-induced ROS accumulation in the testes and contributing to the recovery of sperm motility.

### Penetrability of PCN-222-Mn across BTB

**Fig. 6.**
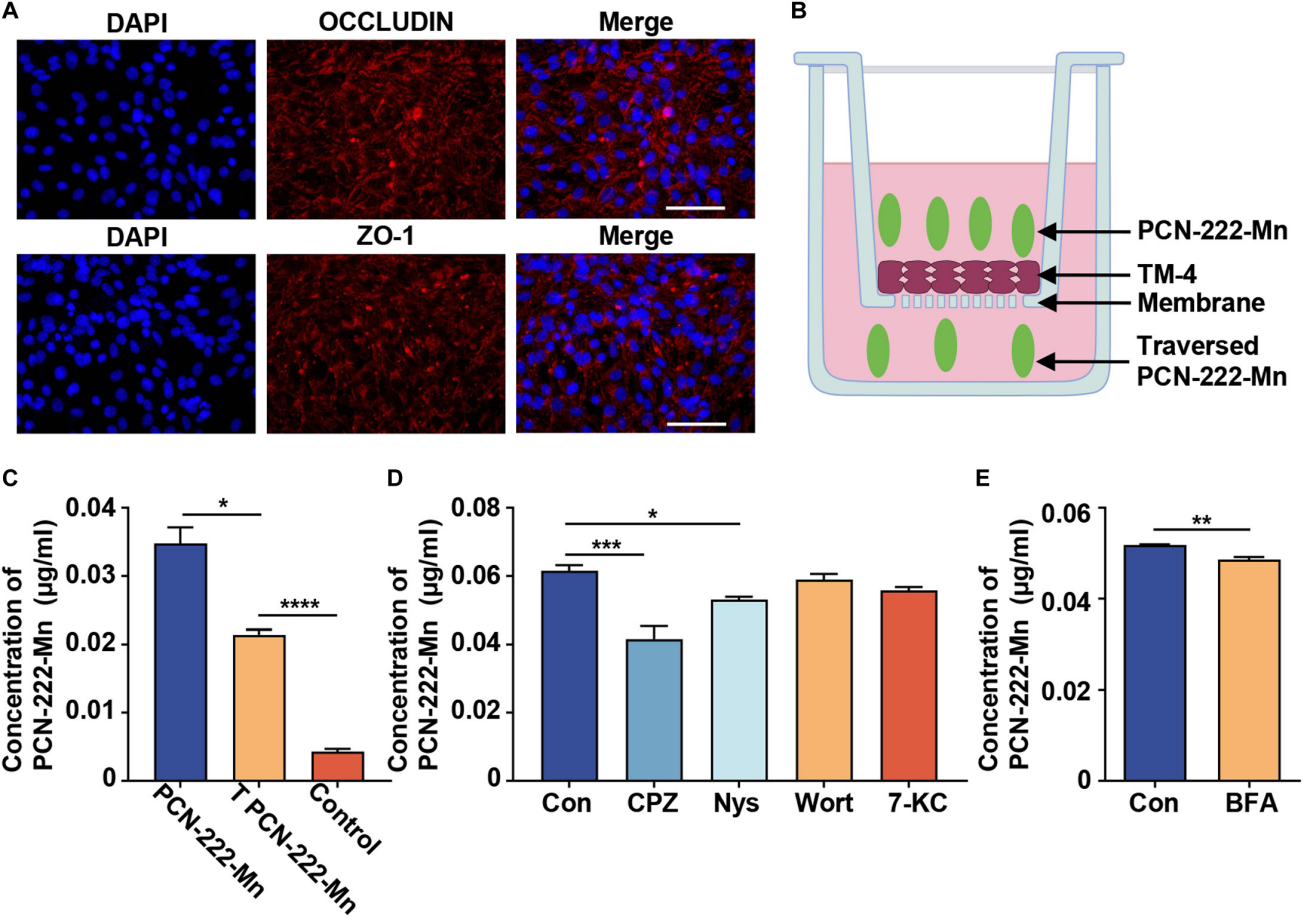
Penetrability of PCN-222-Mn on the blood–testis barrier via Sertoli cell-mediated endocytosis/exocytosis. (A) OCCLUDIN and ZO-1 expression in TM-4 cells. Scale bar: 50 μm. (B) Illustration of the in vitro BTB model. (C) Quantitative analysis the traversed PCN-222-Mn samples and control samples. Data are shown as mean ± SEM, 2-tailed Student’s *t* test for *P* values (*P < 0.05, ****P < 0.0001). (D) Treatment with 1.5 μM CPZ and 10 μM Nys significantly inhibited the uptake of PCN-222-Mn by Sertoli cells. In contrast, treatment of 500 nM Wortmannin and 3.5 μM 7-Ketocholesterol did not produce a significant inhibitory effect on PCN-222-Mn uptake. Data are shown as mean ± SEM, one-way ANOVA for *P* values. (*P < 0.05, ***P < 0.001). (E) Treatment with 10 μg/ml BFA significantly inhibited the exocytosis of PCN-222-Mn. Data are shown as mean ± SEM, 2-tailed Student’s *t* test for *P* values (**P < 0.01).

The BTB is considered one of the tightest junctions in mammals [9], effectively preventing drugs from accessing the testicular environment and exerting their therapeutic effects [10,11,25]. To investigate whether PCN-222-Mn can traverse this barrier, an in vitro model was established using Sertoli cells cultured in a transwell plate. The integrity of the intercellular tight junctions was confirmed by the detection of tight junction proteins ZO-1 and OCCLUDIN, thus validating the formation of a functional in vitro BTB (Fig. 6A).

Following the establishment of this Sertoli cell barrier, PCN-222-Mn was added to the upper chamber and incubated for 24 h to assess its transport (Fig. 6B). Subsequently, medium was collected from both lower and upper chambers. For comparison, a control group consisting of Sertoli cell barriers incubated with vehicle (medium without PCN-222-Mn) in the upper chamber was processed in parallel. Analysis revealed that the PCN-222-Mn concentration in the lower chamber of wells treated with PCN-222-Mn was significantly higher than background levels detected in the lower chamber of control wells. However, this concentration was lower than that remaining in the upper chamber of the PCN-222-Mn-treated wells (Fig. 6C). This indicates that PCN-222-Mn can be transported across the BTB by Sertoli cells, consistent with in vivo results. Additionally, the higher concentration in the upper chamber compared to the lower chamber suggests that the transport mechanism involves endocytosis rather than passive diffusion or paracellular transport. The primary endocytosis pathways include clathrin-mediated endocytosis (CME), caveolae-mediated endocytosis CAV, clathrin-independent/dynamin-independent endocytosis (CLIC/GEEC), fast endophilin-mediated endocytosis (FEME), macropinocytosis, and phagocytosis [26]. To explore the specific cellular uptake and release mechanisms of PCN-222-Mn, we employed inhibitors targeting various endocytosis and exocytosis pathways (Fig. S5A).

The inhibitors used in this study were CPZ, Wort, Nys, 7-KC, and BFA. CPZ disrupts the CME pathway by interfering with the reassembly of AP-2 and clathrin at the cell membrane [27]. Nys targets CAV by impairing cholesterol fluidity within the membrane, thereby hindering endocytic vesicle formation [26]. 7-KC affects the CLIC/GEEC pathway by altering the packing of acyl chains, which impacts membrane fluidity [28]. Wort blocks macropinocytosis by inhibiting PI3K, reducing PIP3 production [29]. BFA disrupts exocytosis by inhibiting Arf1, affecting transport between the Golgi apparatus and the endoplasmic reticulum [30]. To investigate endocytic uptake, established Sertoli cell barriers were pretreated with these inhibitors (CPZ, Nys, 7-KC, and Wort) for 4 h before PCN-222-Mn was added to the upper chamber. After a 24-h incubation with PCN-222-Mn, its concentration in the lower chamber was measured. This experiment showed that pretreatment with CPZ and Nys significantly reduced the amount of PCN-222-Mn transported into the lower chamber compared to barriers exposed to PCN-222-Mn without inhibitors (Fig. 6D). To assess the role of exocytosis in release into the lower chamber, Sertoli cell barriers were first incubated with PCN-222-Mn in the upper chamber for 22 h to allow cellular uptake and initial transport. Subsequently, the medium in the upper and lower chamber was replaced with fresh medium containing BFA (or vehicle for control). After an additional 2 h, the medium from the lower chamber was collected (Fig. S5B). BFA treatment significantly decreased the release of PCN-222-Mn into the lower chamber compared to the control group (Fig. 6E). Collectively, these findings indicate that PCN-222-Mn is internalized by Sertoli cells primarily via CME and CAV endocytosis. Its subsequent release into the lower chamber involves an active exocytosis process. These active transport mechanisms facilitate the effective passage of PCN-222-Mn across the in vitro BTB model.

## Discussion

Chemotherapy poses a serious threat to male reproductive capacity, with risks of both temporary and permanent infertility [31]. The testicular toxicity profiles of chemotherapy drugs depend on many factors, including the patient’s reproductive maturity [31]. In postpubertal and adult patients with active spermatogenesis, chemotherapy directly impairs maturing germ cells and compromises sperm function. For prepubertal patients, these agents can deplete the foundational spermatogonial stem cell pool, threatening future fertility. A common pathogenic mechanism underlying this damage across age groups is the induction of excessive oxidative stress and elevated levels of ROS, which trigger a cascade of testicular injuries. CTX is a commonly used alkylating agent in chemotherapy, and PM is its metabolite. Our study models this clinical scenario using PM in adult mice, demonstrating a strategy to mitigate testicular injury by targeting this shared oxidative pathway. Although physiological ROS concentrations serve critical roles in maintaining normal spermatozoa function, excessive oxidative stress can exceed the cellular capacity for antioxidant protection, ultimately compromising reproductive performance and fertility outcomes. Motility impairment represents one of the most significant consequences of ROS-mediated damage. ROS can compromise sperm membrane integrity [32,33], disrupt regulation of sperm metabolic enzymes [32,34], cause impairments in sperm motility and related cellular signaling cascades [32,35], and induce modifications in sperm DNA [36], which cumulatively contribute to decreased sperm motility. Autophagy is a well-conserved recycling process in cells that becomes activated in response to stressors like increased ROS production [37]. High levels of ROS target essential cellular macromolecules. Through autophagy, cells can uniquely eliminate both oxidized or damaged proteins as well as major ROS-generating organelles including mitochondria and peroxisomes, which serves to mitigate subsequent ROS generation [38,39]. Oxidative modulation of autophagic processes spans its entire pathway: encompassing initiation and phagophore formation through phagophore elongation, autophagosome development, substrate transport to lysosomes, substrate degradation, metabolite recycling, and autophagy-related gene expression. Our study reveals that PCN-222-Mn, via its enzyme-mimicking activity, effectively scavenges ROS and stimulates autophagy, thus maintaining cellular homeostasis and improving sperm motility.

Previous studies have demonstrated that when cultured in vitro until confluence, Sertoli cells can constitute an epithelial monolayer architecture exhibiting both structural and functional characteristics analogous to the native BTB system [40]*.* Our in vitro BTB culture system was employed to ascertain the role of PCN-222-Mn in crossing the BTB. With additional modifications, PCN-222-Mn could potentially enhance its targeting to the testis, which may improve therapeutic efficacy.

From a drug delivery perspective, the PCN-222-Mn platform presented here offers distinct advantages when compared to other established or emerging testicular-targeting strategies, including lipid-based nanoparticles, extracellular vesicles (EVs), and peptide-modified nanoparticles. Lipid-based delivery systems, including liposomes and lipid nanoparticles (LNPs), have achieved substantial clinical success owing to their favorable biocompatibility profiles and physicochemical versatility. Notable examples include PEGylated liposomal doxorubicin for cancer chemotherapy and ionizable LNP-formulated mRNA vaccines for COVID-19 prevention. Nevertheless, these systems face inherent limitations in biodistribution, with unmodified lipid particles being rapidly cleared by the reticuloendothelial system, resulting in high nonspecific accumulation in the liver and spleen [41]. Although PEGylation effectively prolongs circulation by minimizing protein adsorption and immune recognition, it introduces additional challenges including anti-PEG antibody production, accelerated blood clearance phenomenon, and reduced cellular uptake and drug release due to the steric barrier created by PEG chains [42]. Similarly, while EVs hold promise due to their natural biocompatibility, their clinical translation is hindered by low production yields, complex purification requirements, and significant batch-to-batch variability stemming from their dependence on parent cell states, presenting major obstacles to standardized and scalable manufacturing [43]. Another strategy involves peptide-modified nanoparticles, which employ targeting peptides such as homing peptides or cell-penetrating peptides to enhance tissue-specific delivery through recognition of cellular surface markers [44]. However, these systems are constrained by several limitations, including rapid peptide degradation by serum proteases, which reduces their stability in vivo. Additionally, they involve increased manufacturing complexity and cost due to conjugation chemistry and purification processes. Furthermore, dense peptide coverage can cause steric hindrance, impeding drug release and nanoparticle-cell interactions, while potential immunogenic responses may lead to accelerated blood clearance, similar to those observed with PEGylated nanomaterials [45]. In contrast, PCN-222-Mn circumvents many of these issues by leveraging inherent stability, tunable enzymatic activity for targeted oxidative stress modulation, and simplified synthesis that enhances scalability and reduces immunogenicity, thereby positioning it as a promising alternative for testicular delivery applications.

Beyond comparison with alternative nanomaterial platforms, PCN-222-Mn must also be evaluated against conventional antioxidant therapies for male infertility. The clinical efficacy of oral antioxidant supplements has been fundamentally questioned by the recent SUMMER randomized controlled trial. This large-scale study demonstrated that such supplements failed to improve pregnancy outcomes and may even prove detrimental in specific subgroups, particularly among patients with elevated baseline oxidative stress [46]. These limitations stem primarily from their nonspecific systemic delivery, which risks inducing reductive stress, and poor penetration of the BTB to spermatogenesis sites. In contrast, PCN-222-Mn presents a strategy designed to address these fundamental hurdles. Its robust framework provides superior stability against degradation compared to natural supplements or enzymes and critically allows for the incorporation of Mn ions. This feature confers intrinsic MRI contrast properties, enabling noninvasive tracking of its biodistribution and addressing a key limitation of untraceable therapies. Our in vivo imaging confirmed that PCN-222-Mn preferentially accumulates in the testes (Fig. 3E and F) and subsequently traverses the BTB via an active endocytosis mechanism to reach the seminiferous tubules (Fig. 6). Once at the target site, it mitigates oxidative stress through a biomimetic, dual-enzyme catalytic cascade (Fig. 2E and F). This physiological detoxification pathway neutralizes ROS in a controlled manner, thereby avoiding the risk of reductive stress associated with passive antioxidant agents.

Collectively, we have synthesized a nanozyme, PCN-222-Mn, endowed with inherent antioxidant capabilities, which accumulates at high concentrations in the testis. It protects spermatogenesis and sperm from CTX-induced testicular injury by clearing ROS and enhancing autophagy. While PCN-222-Mn exhibits testicular selectivity via MRI (Fig. 3E and F) and BTB penetration (Fig. 6), direct morphological colocalization evidence (e.g., via confocal microscopy to demonstrate colocalization of PCN-222-Mn with clathrin or caveolin-1) would make the endocytic route more definitive in our subsequent research. In addition, we recognize the potential for nanozyme interference with CTX’s ROS-mediated anti-tumor effects. Biodistribution data suggest limited systemic exposure, yet further validation in tumor-bearing models is essential to ensure no compromise to chemotherapy efficacy. Consequently, PCN-222-Mn nanozymes serve as an innovative clinical agent, representing a promising approach to fertility protection of cancer patients.

## Conclusion

In conclusion, this study establishes the synthesized nanozyme PCN-222-Mn as a potent therapeutic agent against CTX-induced testicular injury. PCN-222-Mn safeguards spermatogenesis and sperm function by efficiently scavenging ROS and enhancing cellular autophagy. Its demonstrated testicular accumulation and ability to traverse an in vitro BTB model underscore its capability to reach the target tissue. These findings position PCN-222-Mn as a promising, rationally designed candidate for preserving male fertility in cancer patients undergoing chemotherapy.

## Ethical Approval

The authors are accountable for all aspects of the work in ensuring that questions related to the accuracy or integrity of any part of the work are appropriately investigated and resolved.

## Data Availability

All data needed to evaluate the conclusions in the paper are present in the paper and/or the Supplementary Materials.
