# Supplementary material for: Biomimetic Nanotechnology Overcoming the Blood–Testis Barrier for Testicular Protection in Chemotherapy
Source: Biomater Res. 2026 Jan 30;30:0314. doi: 10.34133/bmr.0314 (PMC12856846; doi:10.34133/bmr.0314)
Supplement: Supplementary 1 — Figs. S1 to S6 Tables S1 and S2 [file bmr.0314.f1.docx]

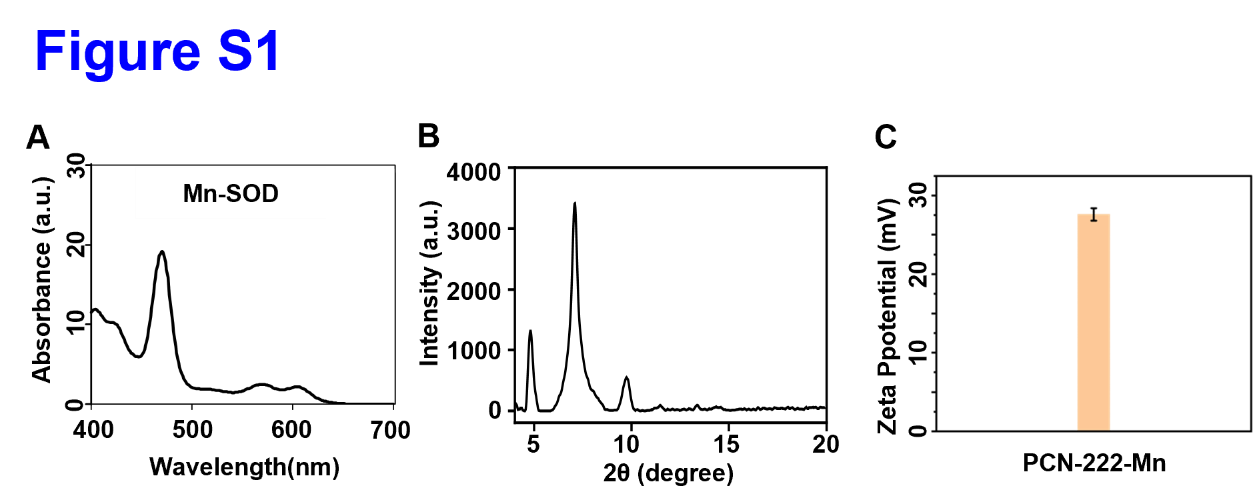


**Figure S1**. (**A**) Absorption spectra of PCN-222-Mn. a.u., arbitrary units. (**B**) Powder x-ray diffraction (PXRD) patterns of PCN-222-Mn. (**C**) Zeta potential distribution of PCN-222-Mn.


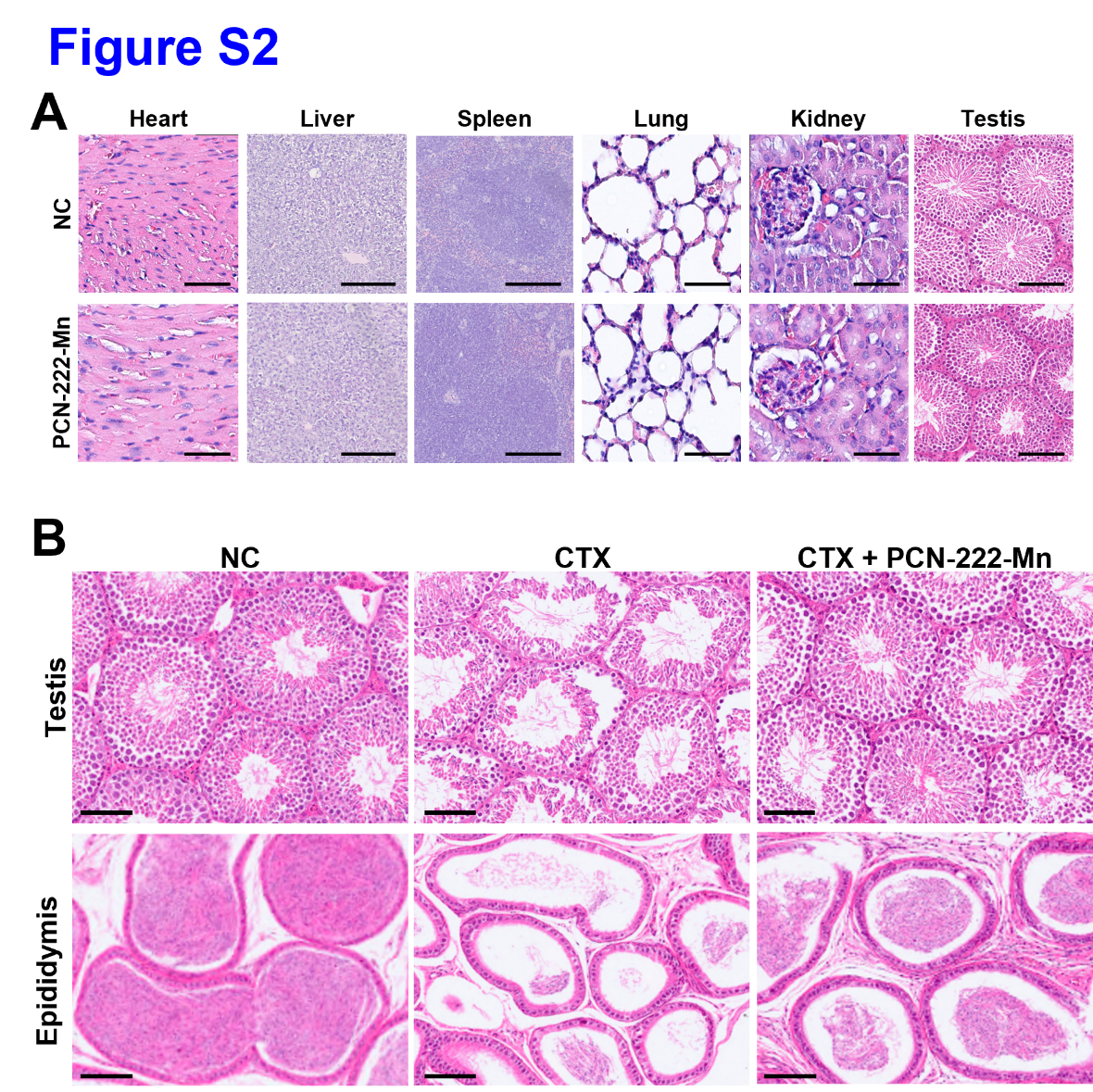


**Figure S2**. (**A**) Biocompatibility of PCN-222-Mn in vivo. H&E staining of heart, liver, spleen, lungs, kidneys, and testes 24 hours after PCN-222-Mn injection. Scale bar: 200 μm. (**B**) H&E staining of testis and cauda epididymis. Scale bar: 100 μm.


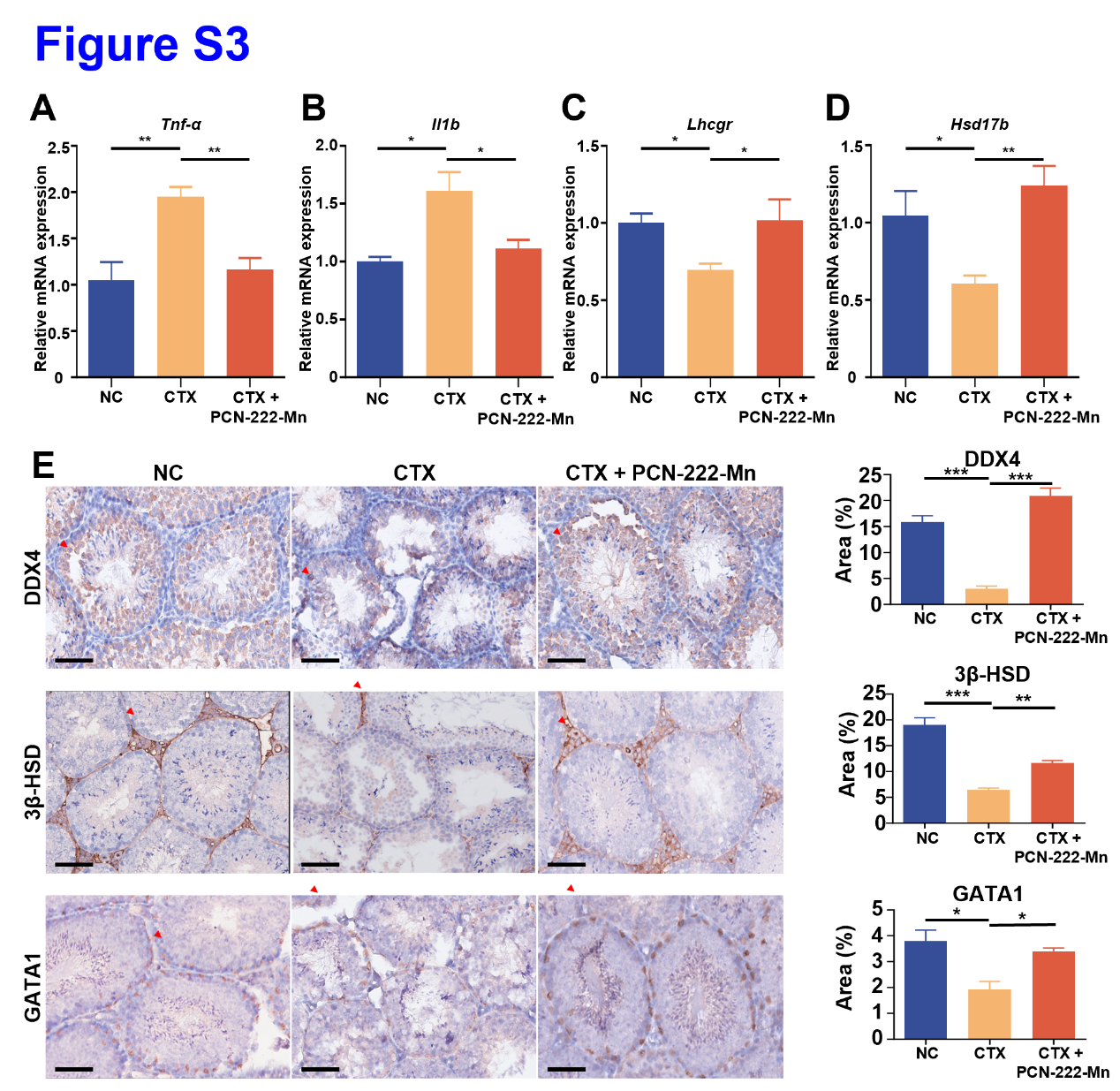


**Figure S3**. Rescued expression of testicular genes and proteins after PCN-222-Mn treatment. (**A-D**) Relative mRNA expression of Tnf-α (A), Il1b (B), Lhcgr (C), and Hsd17b (D) in testis. (**E**) Immunohistochemical staining and statistical analysis of DDX4, 3β-HSD, and GATA1 in testis sections. Scale bar: 100 μm. Data are shown as mean ± SEM, two-tailed Student’s t-test for P values (*: P < 0.05, **: P < 0.01, ***: P < 0.001).


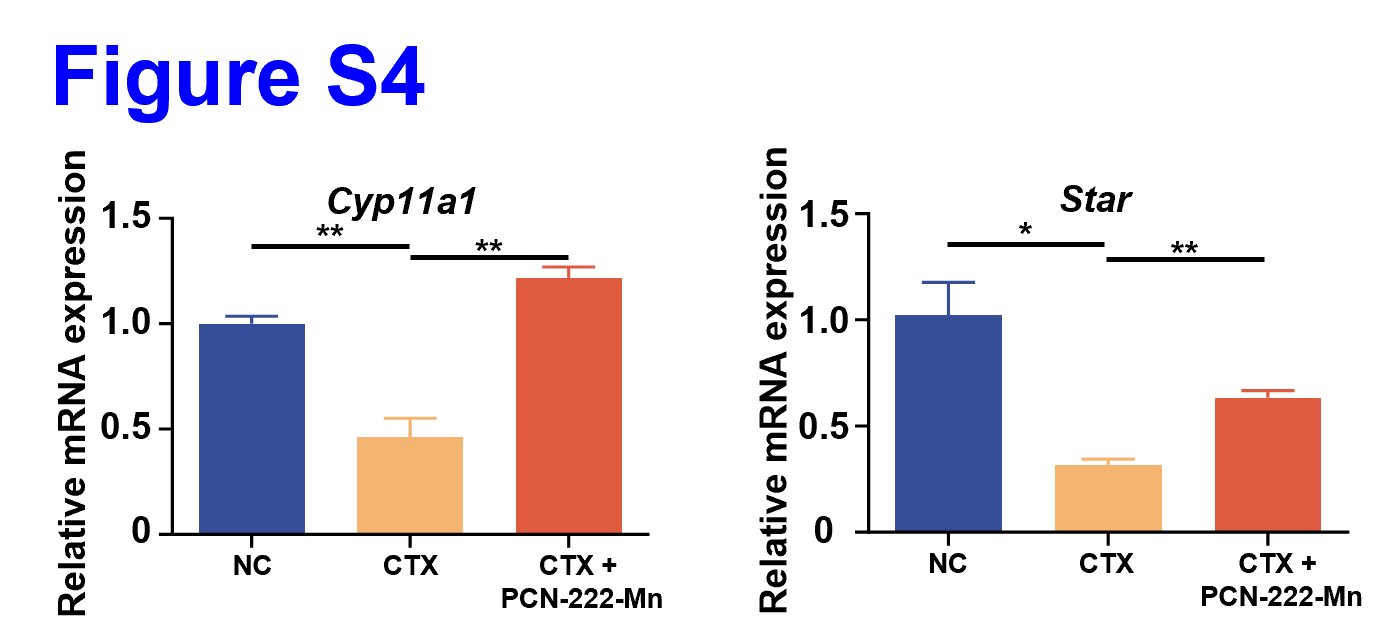


**Figure S4**. Relative mRNA expression of Cyp11a1, and Star in testis. Data are shown as mean ± SEM, two-tailed Student’s t-test for P values (*: P < 0.05, **: P < 0.01).


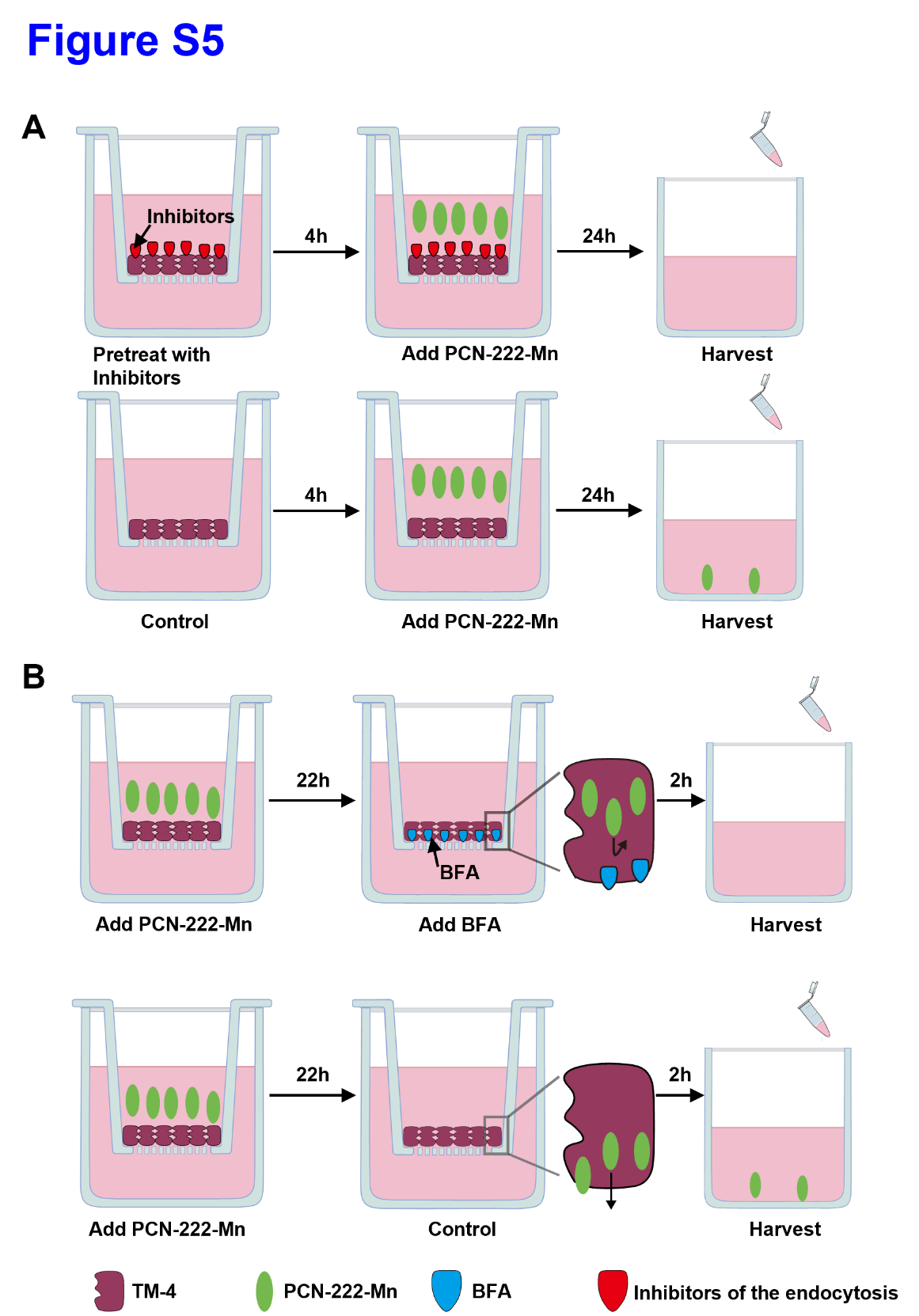


**Figure S5**. (**A**) Endocytosis inhibitors prevented PCN-222-Mn from crossing the BTB, while transport occurred in control conditions. (B) Exocytosis inhibition with BFA resulted in PCN-222-Mn accumulation at the upper chamber, while successful crossing was observed without inhibitors.


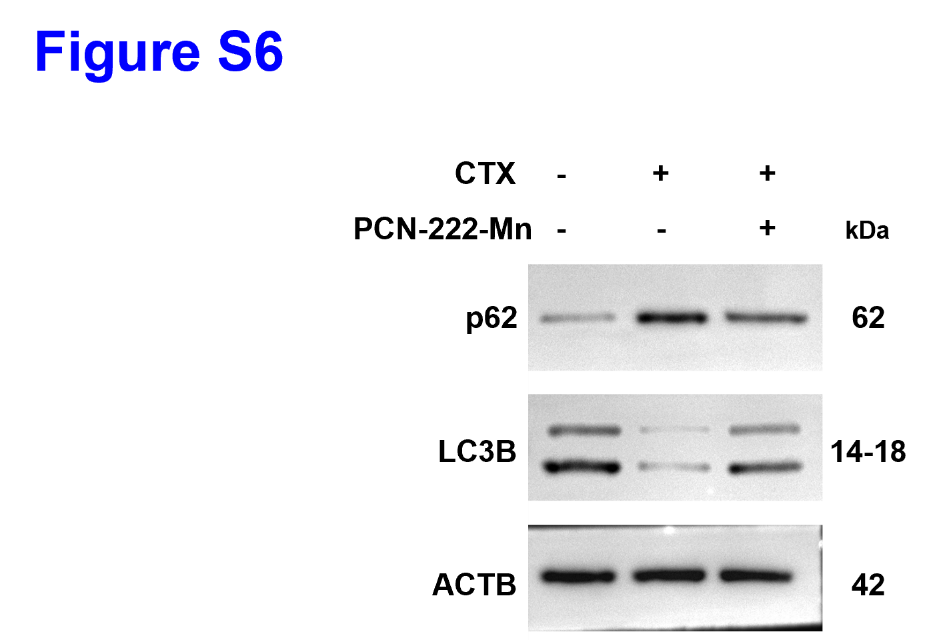


**Figure S6.** Western blot analysis of autophagic flux markers in testicular tissues.

**Table S1** Power analysis for sample size determination in CTX-induced testicular injury model.

| Outcome Measures^a^ | Comparison | Effect Size^b^ | Minimum n per Group^c^ |
| --- | --- | --- | --- |
| Sperm concentration | NC vs. CTX | Cohen's d = 5.98 | 2 |
|  | CTX vs. Treatment | Cohen's d = 2.54 | 4 |
| Sperm motility (%) | NC vs. CTX | Cohen's d = 4.39 | 3 |
|  | CTX vs. Treatment | Cohen's d = 2.36 | 5 |

^a^Outcome measures were selected and effect sizes derived based on a previous similar study (Zhao et al., 2024).

^b^Cohen's d represents the standardized mean difference; values >0.8 indicate large effects.

^c^Minimum n per group was calculated using G*Power 3.1.9.7 as the smallest integer required to achieve 80% power (1−β = 0.80) at α = 0.05 (two-tailed independent t-test) based on estimated Cohen's d.

**Table S2** Primer sequence of the target gene.

| Gene | Forward (5'-3') | Reverse (5'-3') |
| --- | --- | --- |
| SOD2 | CAGACCTGCCTTACGACTATGG | CTCGGTGGCGTTGAGATTGTT |
| CAT | AGCGACCAGATGAAGCAGTG | TCCGCTCTCTGTCAAAGTGTG |
| LhcgR | CGCCCGACTATCTCTCACCTA | GACAGATTGAGGAGGTGTCAAA |
| CYP11A1 | AGGTCCTTCAATGAGATCCCTT | TCCCTGTAAATGGGGCCATAC |
| FSHR | CCTTGCTCCTGGTCTCCTTG | CTCGGTCACCTTGCTATCTTG |
| HSD17β3 | AGGTTCTCGCAGCACCTTTT | CATCGCCTGCTCCGGTAATC |
| Star | ATGTTCCTCGCTACGTTCAAG | CCCAGTGCTCTCCAGTTGAG |
| IL-1β | GAAATGCCACCTTTTGACAGTG | TGGATGCTCTCATCAGGACAG |
| TNF | CAGGCGGTGCCTATGTCTC | CGATCACCCCGAAGTTCAGTAG |
| ACTB | GGCTGTATTCCCCTCCATCG | CCAGTTGGTAACAATGCCATGT |
